# Hypomethylation of Interleukin-6 Promoter is Associated with the Risk of Coronary Heart Disease

**DOI:** 10.5935/abc.20160124

**Published:** 2016-08

**Authors:** Hong-Peng Zuo, Ying-Yu Guo, Lin Che, Xian-Zheng Wu

**Affiliations:** 1 Department of Emergency, Tongji Hospital, Tongji University, Shanghai, China; 2 Department of Computed Tomography, Jilin Oilfield General Hospital, Songyuan, Jilin Province, China; 3 Department of Cardiology, Tongji Hospital, Tongji University, Shanghai, China

**Keywords:** Coronary Artery Disease, Interleukin-6, DNA Methylation, Epigenetic Repression

## Abstract

**Background:**

Interleukin-6 (IL-6) is implicated in the pathogenesis of coronary heart disease (CHD), and IL-6 expression has associated with reduced DNA methylation of its gene promoter. However, there are no data on IL-6 promoter methylation and the risk of CHD.

**Objective:**

To examine whether IL-6 promoter methylation measured in blood leukocyte DNA is associated with CHD risk.

**Methods:**

A total of 212 cases with CHD and 218 controls were enrolled. Methylation at two CpG sites in IL-6 promoter was measured by bisulfite pyrosequencing, and the mean IL-6 methylation was calculated by averaging the methylation measures of the two CpGs.

**Results:**

Mean methylation level in IL-6 promoter in CHD cases was significantly lower than that in controls (p = 0.023). Logistic regression analysis showed that IL-6 methylation was inversely associated with the risk of CHD. The odds ratios (ORs) of CHD for subjects in the second and first (lowest) tertile of IL-6 methylation were 1.87 (95% CI = 1.10‑3.20) and 2.01 (95% CI = 1.19-3.38) (p_trend_ = 0.013), respectively, compared to subjects in the third (highest) tertile. The IL-6 hypomethylation-related risk estimates tended to be stronger for acute myocardial infarction (p_trend_ = 0.006). CpG position-specific analysis showed that hypomethylation of position 1 conferred a more pronounced increase in CHD risk than that of position 2.

**Conclusion:**

These findings suggest that DNA hypomethylation of IL-6 promoter is associated with the increased risk for CHD, especially for acute myocardial infarction. The two distinct CpGs in IL-6 may contribute differently to the development of CHD.

## Introduction

DNA methylation is an epigenetic modification that plays a crucial role in controlling gene expression in the genome.^1^ In mammals, DNA methylation involves addition of methyl groups to cytosine of a CpG dinucleotide to form 5-methylcytosine (5 mC). Epigenetic changes in methylation patterns are increasingly implicated in a number of human diseases.^2^ Aberrant promoter methylation of several genes has been associated with the development and progression of coronary heart disease (CHD).^3-8^

The major cause of CHD is atherosclerosis, an inflammatory disease of the arteries associated with lipid and other metabolic alterations. Interleukin-6 (IL-6) is central to inflammatory processes underlying chronic inflammatory diseases including CHD,^9-11^ and have been shown to induce the expression of other genes that might contribute to the CHD phenotype.^12^ Genetic variant in IL-6 gene promoter has been associated with its abnormal expression^13,14^ and increased risk of CHD.^15^ Methylation modification is an alternative mechanism regulating IL-6 production,^16,17^ and consistent evidence has shown that IL-6 expression is associated with reduced DNA methylation of its gene promoter.^18-24^ In addition, DNA methylation in IL-6 promoter has been associated with risk factors for CHD such as air pollution exposure.^25^ However, to the best of our knowledge, there are no data on IL-6 promoter methylation and the risk of CHD.

Recently, gene silencing by DNA methylation has been suggested to be associated with methylation not only in CpG islands, but also in CpG island shores (i.e., on the island edges).^26,27^ In the present study on the association between IL-6 promoter methylation and CHD risk, we focused on two individual CpGs within a CpG shore located in the IL-6 gene promoter region, which were described to be associated with lung function and air pollution exposure.^25,28^

## Methods

### Study participants

A total of 212 patients with CHD, including 120 cases of acute myocardial infarction (AMI), 42 cases of prior myocardial infarction and 50 cases of unstable angina, were recruited from Tongji Hospital, Tongji University between January 2011 and June 2012. The diagnosis of CHD was established by angiographic evidence of ≥ 70% stenosis of 1 major coronary artery, and/or ≥ 50% of the left main coronary artery. As control, 218 CHD-free subjects, determined by history analysis, physical examination, electrocardiography, and echocardiography were recruited from the same hospital during the same period when the case patients were recruited. The controls were frequency-matched to the cases by age (±5 years) and sex. For both CHD and control groups, subjects with hypertension, diabetes, peripheral artery disease, autoimmune-related disease or cancers were excluded. Information on age, sex, height, weight, cigarette smoking, and family history of CHD was obtained using structured questionnaire through in-person interviews. Body mass index (BMI) was calculated using the formula: body weight in kilograms divided by the square of body height in meters (kg/m^2^). An ever-smoker was defined as a smoker of at least 1 cigarette per day for at least 6 months. Information on serum total cholesterol was collected on the basis of medical records. Written informed consent was obtained from all participants. This study was conducted with approval from the Ethics Committee of Tongji Hospital (E20100401).

### DNA methylation analysis of IL-6 promoter

Peripheral blood leukocytes were isolated by Ficoll-Hypaque density gradient centrifugation. DNA was extracted from leukocytes using the QIAamp DNA Blood kit (Qiagen, Shanghai, China), and then bisulfite-converted with the Zymo EZ DNA Methylation kit (Zymo, CA, USA). PCR-based pyrosequencing was performed to quantitate methylation of the IL-6 promoter. PCR to amplify the target region covering the two CpGs [Genbank Accession no.M18403, chromosome 7: 22733847 (position1) and chromosome 7: 22733841 (position 2)] was carried out in a 50 µl reaction volume containing 25 µl of GoTaq Master mix (Promega, WI, USA), 10 pmol of biotinylated forward primer (biotin-TAT TTT AGT TTT GAG AAA GGA GGT G), 10 pmol of reverse primer (CAA TAC TCT AAA ACC CAA CAA AAA C), and 50 ng of bisulfite-treated genomic DNA. The cycling program was 5 min at 95°C followed by 45 cycles of each 95°C for 1 min, 57°C for 1 min and 72°C for 1 min and a final elongation for 5 min at 72 °C. Then, sequencing was performed on the PSQ HS 96 Pyrosequencing System using 0.3 µM pyrosequencing primer (TCC TAA TAC AAA CAA CCC C). Non-CpG cytosine residue was used as built-in control to verify bisulfite conversion. The degree of methylation was expressed for each CpG as %5 mC (%5 mC) over the sum of methylated and unmethylated cytosines. %5 mC levels of the two CpGs were averaged to obtain a mean methylation measure of the IL-6 promoter.

### Statistical analysis

Differences in age (< 65, ≥ 65 years), sex, BMI (< 24, 24-27.9, ≥ 28 kg/m^2^), ever-smoker (no, yes), serum total cholesterol (< 4.67, 4.67-5.47, > 5.47 mmol/L) and family history of CHD (no, yes) between CHD cases and controls were evaluated using χ^2^-test. Tertile cut-points of mean, position 1 and position 2 methylation measures in IL-6 promoter were based on the values among controls. The associations of IL-6 methylation with CHD risk were estimated by computing the odds ratios (ORs) and 95% confidence intervals (CIs) from multivariate logistic regression analyses with adjustment for BMI (< 24, 24-27.9, ≥ 28 kg/m^2^), serum total cholesterol (< 4.67, 4.67-5.47, > 5.47 mmol/L) and family history of CHD (no, yes). Further adjustment by age (< 65, ≥ 65 years), sex and ever-smoker (no, yes) did not materially alter the risk estimates and thus these variables were not included in the final models. All tests were two-sided and a p value of less than 0.05 was considered significant. Data was analyzed with Stata 10.1 software (Stata Corporation, College Station, TX).

## Results

### Characteristics of the study subjects

The characteristics of the CHD cases and controls are shown in Table 1. No significant differences between cases and controls were observed in the distributions of age, sex, or ever-smoker. Cases with CHD were more likely to have higher BMI (p = 0.029), higher serum total cholesterol (p < 0.001) and frequent family history of CHD (p < 0.001) than controls.

**Table 1:** Characteristics of coronary heart disease (CHD) cases and control subjects.

**Characteristic**	**Controls, n (%)**	**CHD cases, n (%)**	p value*
**Age (years)**			
< 65	102 (46.8)	108 (50.9)	
≥ 65	116 (53.2)	104 (49.1)	0.389
**Sex**			
Female	46 (21.1)	48 (22.6)	
Male	172 (78.9)	164 (77.4)	0.699
**Body mass index (kg/m^2^)**			
< 24	125 (57.3)	96 (45.3)	
24-27.9	61 (28.0)	69 (32.5)	
≥ 28	32 (14.7)	47 (22.2)	0.029
**Ever-smoker**			
No	100 (45.9)	79 (37.3)	
Yes	118 (54.1)	133 (62.7)	0.070
**Serum total cholesterol (mmol/L)**			
< 4.67	73 (33.5)	34 (16.1)	
4.67-5.47	75 (34.4)	52 (24.5)	
> 5.47	70 (32.1)	126 (59.4)	< 0.001
**Family history of CHD**			
No	214 (98.2)	185 (87.3)	
Yes	4 (1.8)	27 (12.7)	< 0.001

* p value obtained from a χ2-test comparing cases and Controls.

### Association between mean IL-6 methylation and CHD risk

**Figure 1:**
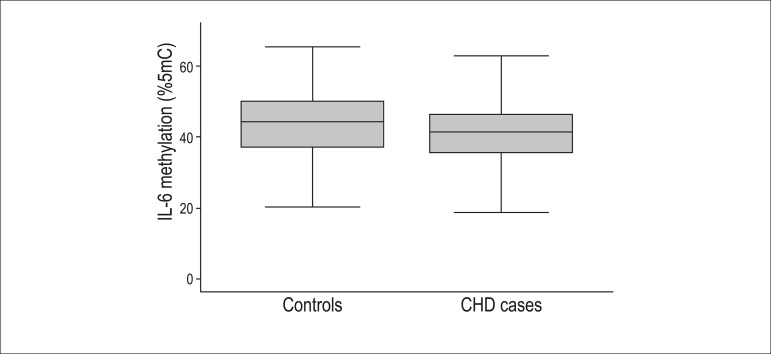
Comparison of IL-6 methylation levels measured in blood leukocyte in coronary heart disease (CHD) cases and controls (p = 0.023).

CHD cases had significantly reduced mean IL-6 methylation level than controls (mean (standard deviation, SD): 41.2 (0.7) versus 43.4 (0.6), p = 0.023, Figure 1). Logistic regression analysis showed that IL-6 promoter methylation was inversely associated with the risk of CHD (Table 2). The ORs of CHD for subjects in the second and first (lowest) tertile of mean IL-6 methylation were 1.87 (95% CI = 1.10-3.20) and 2.01 (95% CI = 1.19-3.38) (p_trend_ = 0.013), respectively, compared to individuals in the third (highest) tertile. When evaluated by clinical types of CHD, the IL-6 hypomethylation-related risk estimates tended to be stronger for AMI, with OR of 2.00 (95% CI = 3.2-5.2) for the second tertile and 2.57 (95% CI = 1.33-4.95) for the first tertile (p_trend_ = 0.006, Table 2).

**Table 2:** Association of IL-6 promoter methylation with risk of coronary heart disease (CHD).

**Methylation (%5 mC)**	**Controls**	**CHD cases**							
		**All**	**AMI**			**Prior MI**		**Angina**	
	**n (%)**	**n (%)**	OR (95% CI)*	**n (%)**	OR (95% CI)*	**n (%)**	OR (95% CI)*	**n (%)**	OR (95% CI)*
**Mean (Tertile†)**									
T3 (> 47.7)	73 (33.5)	42 (19.8)	1.0 (reference)	21 (17.5)	1.0 (reference)	9 (21.4)	1.0 (reference)	12 (24.0)	1.0 (reference)
T2 (40.1-47.7)	71 (32.6)	77 (36.3)	1.87 (1.10-3.20)	41 (34.2)	2.00 (1.01-3.94)	18 (42.9)	2.02 (0.84-4.86)	18 (36.0)	1.76 (0.73-4.24)
T1 (< 40.1)	74 (33.9)	93 (43.9)	2.01 (1.19-3.38)	58 (48.3)	2.57 (1.33-4.95)	15 (35.7)	1.63 (0.66-4.01)	20 (40.0)	1.83 (0.77-4.33)
			*p*-trend=0.013		*p*-trend=0.006		*p*-trend=0.325		*p*-trend=0.182
**Position 1 (Tertile†)**									
T3 (> 51.7)	73 (33.5)	44 (20.8)	1.0 (reference)	25 (20.8)	1.0 (reference)	8 (19.1)	1.0 (reference)	11 (22.0)	1.0 (reference)
T2 (43.5-51.7)	71 (32.6)	77 (36.3)	2.04 (1.20-3.49)	38 (31.7)	1.71 (0.88-3.32)	19 (45.2)	2.37 (0.96-5.84)	20 (44.0)	2.50 (1.01-6.17)
T1 (< 43.5)	74 (33.9)	91 (42.9)	2.17 (1.29-3.66)	57 (47.5)	2.44 (1.29-4.62)	15 (35.7)	1.94 (0.77-4.92)	19 (38.0)	2.16 (0.88-5.27)
			*p*-trend=0.05		*p*-trend=0.006		*p*-trend=0.196		*p*-trend=0.114
**Position 2 (Tertile†)**									
T3 (> 44.0)	73 (33.5)	51 (24.1)	1.0 (reference)	30 (25.0)	1.0 (reference)	9 (21.4)	1.0 (reference)	12 (24.0)	1.0 (reference)
T2 (35.9-44.0)	71 (32.6)	63 (29.7)	1.25 (0.74-2.12)	28 (23.3)	0.93 (0.48-1.81)	17 (40.5)	2.06 (0.84-5.02)	18 (36.0)	1.74 (0.72-4.20)
T1 (< 35.9)	74 (33.9)	98 (46.2)	1.73 (1.05-2.86)	62 (51.7)	1.81 (0.99-3.29)	16 (38.1)	1.82 (0.74-4.47)	20 (40.0)	1.79 (0.76-4.23)
			*p*-trend=0.030		*p*-trend=0.038		*p*-trend=0.220		*p*-trend=0.197

%5mC: percentage of 5-methylcytosine; AMI: acute myocardial infarction; CI: confidence interval; MI: myocardial infarction; OR: odds ratio.

* Adjusted by body mass index (<24, 24-27.9, ≥28 kg/m2), serum total cholesterol (<4.67, 4.67-5.47, >5.47 mmol/L) and CHD family history (no, yes).

† The tertiles of IL-6 methylation measures were based on values among control subjects.

### CpG position-specific association between IL-6 methylation and CHD risk

Our main analysis considered the mean methylation of the two CpGs in IL-6 promoter. However, methylation at specific positions within a gene's promoter may affect gene expression differently. Therefore, we further examined the associations between position-specific methylation in IL-6 and CHD risk. As shown in Table 2, although significant p_trend_ values for CHD and AMI were observed for both CpG positions, hypomethylation of position 1 conferred a more pronounced increase in CHD risk when compared to that of position 2.

## Discussion

Promoter methylation is an essential epigenetic mechanism for the regulation of the IL-6 expression.^16,17^ It has been reported that hypomethylation of IL-6 promoter was associated with the pathogenesis of systemic lupus erythematosus, rheumatoid arthritis and chronic periodontitis.^18,24,29^ In the present study, we demonstrated for the first time that DNA hypomethylation in IL-6 promoter was associated with increased risk for CHD, especially for AMI. These data suggest that demethylation of the IL-6 promoter might be a common epigenetic basis for the development of a variety of inflammation-associated diseases. In this context, the associations between IL-6 promoter methylation and the risk of other inflammatory diseases merit further investigations.

Methylation level of IL-6 promoter has been associated with air pollution exposure,^25^ which has been known to increase cardiovascular morbidity and mortality.^30^ In addition, serum IL-6 level has been associated with increased risk of mortality in patients with CHD.^31^ In the present study, we observed that hypomethylation in IL-6 promoter was associated with a stronger risk estimate for AMI, a clinical type of CHD contributing to large cases of mortality worldwide.^32^ Based on these preliminary results and the current literature, it is tempting to speculate that demethylation of the IL-6 promoter may play a role not only in the development, but also in the prognosis of CHD. In line with this hypothesis, the methylation level of the IL-6 promoter has been reported to be significantly correlated to the development and the severity of systemic lupus erythematosus.^18,24^

There is increasing evidence that methylation at specific positions within a gene's promoter may be more important for gene expression than the mean methylation of CpG sites in the promoter region.^33,34^ In the present study, we therefore further examined whether the CHD associations we observed in the main analysis using mean methylation were specific to certain positions in the IL-6 promoter. We observed that hypomethylation of the CpG at position 1 in IL-6 promoter conferred a more pronounced increase in CHD and AMI risk estimates than that at position 2. Previously, methylation levels of these two CpGs in IL-6 have been differently associated with air pollution exposure.^25^ These data suggest that differential DNA hypomethylation of the two distinct CpGs in IL-6 may reflect different cumulative effects from endogenous and exogenous exposure factors, and then contribute differently to the susceptibility to human diseases including CHD. Transcription factor binding sites (BAF155, Inil, c-Myc, BAF170, Max, NRSF and Nrf1) were present for position 1, whereas position 2 was free of the binding sites,^28^ which may partly explain the different results we and others observed for these two positions. Further studies are required on whether the different CpGs in IL-6 promoter have differential effects on IL-6 expression.

Our study was based on accurate quantitative analysis using pyrosequencing methodology, which is suitable for measuring subtle changes in DNA methylation and can produce individual measures of methylation at more than one CpG site, thus reflecting more accurately DNA methylation in the region.^35,36^ However, several limitations of the present study should be noted. Firstly, while our data demonstrate that IL-6 promoter hypomethylation was associated with CHD, whether the methylation pattern is a cause or a consequence of the development of CHD cannot be determined in our case-control study design. Prospective studies are required to elucidate the temporal nature of this association. However, given the implication of IL-6 in the pathogenesis of CHD and the inverse correlation of IL-6 promoter methylation with CHD risk factors,^25,37^ our results suggest that demethylation of the IL-6 promoter may contribute to the risk of developing CHD. Secondly, the functional effect of the IL-6 promoter hypomethylation was not further investigated in the present study. Finally, several studies have observed correlations of DNA methylation in IL-6 promoter with diet and environmental exposures,^25,38-40^ which would confound the associations between IL-6 promoter hypomethylation and risk of CHD that we observed.

## Conclusion

In summary, although limited by relatively small sample size, the present study suggest that DNA hypomethylation in IL-6 promoter is associated with the increased risk for CHD, especially for AMI. Demethylation of the two CpGs in IL-6 may contribute differently to the susceptibility to CHD. Because of the exploratory nature of the present study, future studies will be needed to verify our findings.
